# Multi-Modal Regulation of Circadian Physiology by Interactive Features of Biological Clocks

**DOI:** 10.3390/biology11010021

**Published:** 2021-12-24

**Authors:** Yool Lee, Jonathan P. Wisor

**Affiliations:** Department of Translational Medicine and Physiology, Elson S. Floyd College of Medicine, Washington State University, Spokane, WA 99202, USA; j_wisor@wsu.edu

**Keywords:** circadian clock, circadian disruption, SCN, brain clocks, peripheral clocks, redox metabolism

## Abstract

**Simple Summary:**

Circadian rhythms, driven by molecular clockwork, exist in nearly all cells and tissues throughout the body, and these rhythmic oscillators constitute tightly coupled network systems that generate rhythmic physiology and behavior. Common circadian disrupting factors, such as night shift work and mistimed eating, can increase the risk of disease onset and symptoms. Disease can then, in turn, promote circadian disruption, leading to a vicious cycle of adverse circadian and overall health effects. This relationship suggests the importance of well-coordinated internal clock systems that maintain optimal synchrony with environmental and metabolic cues. In this review, we will recount the recent advances in circadian clock research and discuss how circadian clocks reciprocally interact with other signaling and metabolic factors to coordinate the daily rhythms of physiology.

**Abstract:**

The circadian clock is a fundamental biological timing mechanism that generates nearly 24 h rhythms of physiology and behaviors, including sleep/wake cycles, hormone secretion, and metabolism. Evolutionarily, the endogenous clock is thought to confer living organisms, including humans, with survival benefits by adapting internal rhythms to the day and night cycles of the local environment. Mirroring the evolutionary fitness bestowed by the circadian clock, daily mismatches between the internal body clock and environmental cycles, such as irregular work (e.g., night shift work) and life schedules (e.g., jet lag, mistimed eating), have been recognized to increase the risk of cardiac, metabolic, and neurological diseases. Moreover, increasing numbers of studies with cellular and animal models have detected the presence of functional circadian oscillators at multiple levels, ranging from individual neurons and fibroblasts to brain and peripheral organs. These oscillators are tightly coupled to timely modulate cellular and bodily responses to physiological and metabolic cues. In this review, we will discuss the roles of central and peripheral clocks in physiology and diseases, highlighting the dynamic regulatory interactions between circadian timing systems and multiple metabolic factors.

## 1. Introduction

Since the formation and settlement of our solar system roughly 4.5 billion years ago, the Earth has been rotating on its axis and revolving around the Sun, bringing about continuous light and dark cycles on this planet. In accordance with these cycles, nearly all living unicellular and multicellular organisms have evolved rhythmic life processes controlled by the circadian clock (from the Latin phrase *circa diem* meaning “about a day”), which have about 24 h periods and constantly predict and adapt to daily environmental changes [1]. This internal timing system is believed to help organisms survive by increasing their ability to timely anticipate the cyclic changes of light and food availability as well as predation risk. At the unicellular level, the timely compartmentalization of the organism’s biochemical, metabolic, and redox processes, coordinated by the intrinsic clockwork, ensures the temporal fitness of cell physiology and functions across species [2]. In lower eukaryotic species, biological timers may help single-celled organisms escape the DNA-damaging effects of ionizing radiation from sunlight as well as oxidative stress during cell division [3]. In higher, complex organisms, including humans, common cellular oscillators constitute the brain and peripheral tissue clocks that interconnect to form a circadian network at the whole-body level. Interestingly, human or cross-species studies reveal differences in behavioral chronotype not only between species but also within and between different individuals of each species, suggesting the extent of intra- and inter-subject variability of circadian timing systems [4,5,6,7,8]. With periodic anticipation and synchronization to day–night cycles, these internal timing systems, in concert, coordinate rhythmic physiology and behaviors such as the sleep–wake cycle, body temperature, blood pressure, hormone production, neural and immune system processes, and cell proliferation [9,10].

In contrast to the adaptive benefits of circadian rhythms, modern lifestyles result in misalignment of the working, eating, and sleeping cycles, relative to natural 24 h light/dark cycles, that historically defined human existence. Such misalignment has been found to increase our susceptibility to the onset and development of cardiometabolic, digestive, immune, and neuropsychiatric disorders, as well as cancers [11,12,13,14]. Several studies in animal models containing genetic mutations of clock genes or those exposed to forced circadian desynchrony regimens have also reinforced the causal relationship between circadian disturbances and disease pathologies [15,16,17,18,19,20]. Furthermore, increasing numbers of studies in cellular and animal models have reported that cellular and bodily rhythms are highly influenced by physiological and metabolic stimuli, such as diet, exercise, metabolites, ions, and gaseous molecules [21,22,23]. In this review, we will describe recent advances in chronobiology as well as the roles of central and peripheral clocks in physiology and diseases, with a particular focus on the dynamic interactions between biological timing systems and metabolic factors.

## 2. Multi-Modal Mechanisms of Circadian Physiology

**Figure 1:**
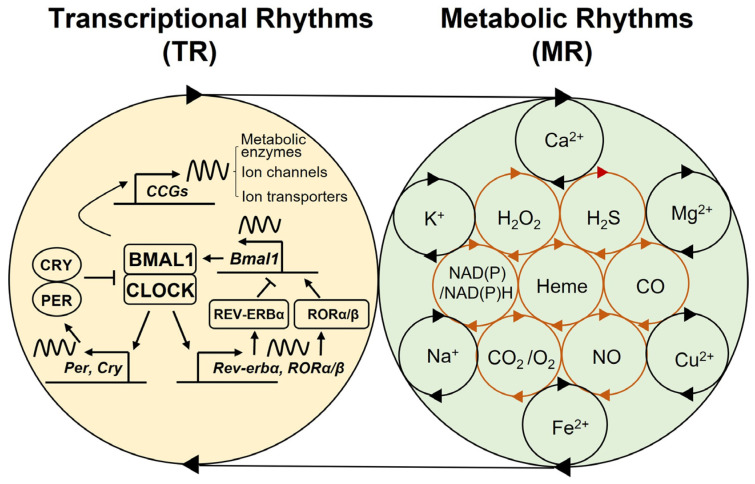
Coupled-cellular oscillators. Bidirectional regulation between transcriptional and metabolic rhythms. The auto-regulatory feedback cycles between the CLOCK/BMAL1 transcriptional activator complex and its transcriptional repressors (PER/CRY, REV-ERBα) and activators (RORα/β), constituting the molecular clock oscillator. This oscillator drives the expression of multiple clock-controlled genes (CCGs), such as metabolic enzymes, ion channels, and transporters. The transcriptional rhythms (TR) mediate metabolic rhythms (MR) involving the cyclic synthesis, degradation, and transport (e.g., influx/efflux) of redox factors, gases, and ions, which, in turn, provide feedback that regulates the TR, constituting coupled-cellular oscillators.

The basic circadian rhythm mechanism, conserved across living species on earth, is typically characterized by a cell-autonomous autoregulatory feedback loop [3,24]. In eukaryotes, a subset of dedicated positive and negative clock regulators forms the interlocked transcriptional translational feedback loop (TTFL), which constitutes a cell-intrinsic oscillator that drives the rhythmic expression of output genes involved in metabolic, biosynthetic, and signal transduction pathways [9]. In mammals, the BMAL1 and CLOCK transcriptional activator complex cyclically drives the transcription of its own repressors, period (PER) and cryptochrome (CRY). The core oscillator is complemented by a second loop in which periodic expression of *BMAL1* is maintained by the REV-ERBα/β repressor and RORα/β activator proteins [25] (Figure 1).

Besides the core regulatory loops, multiple levels of epigenetic, posttranscriptional, and posttranslational regulation involving various kinases and phosphatases, ubiquitin–proteasome pathway components, nuclear–cytoplasmic transporters, non-coding RNAs, and chromatin remodelers contribute to the molecular clockwork, thus coordinating temporal programs via multiple clock–output genes involved in cellular physiology and metabolism [26,27,28,29,30,31,32,33]. Notably, recent large-scale genomic studies reveal that ~50% of mammalian genes exhibit circadian regulation in at least one tissue, although the identity of genes expressed rhythmically varies from tissue to tissue [34,35,36]. In addition, multi-scale omics studies demonstrated circadian regulation of the epigenome, the metabolome, the proteome/phosphoproteome, and the microbiome [34,35,37,38,39,40,41,42,43,44,45]. These studies reveal that proteins or metabolites display different patterns of oscillations relative to transcript rhythms in a given tissue (e.g., hippocampus, liver), and oscillations at all levels can be reprogramed by circadian disturbances, such as sleep deprivation, jet lag, high-fat diet, and aging.

**Figure 2:**
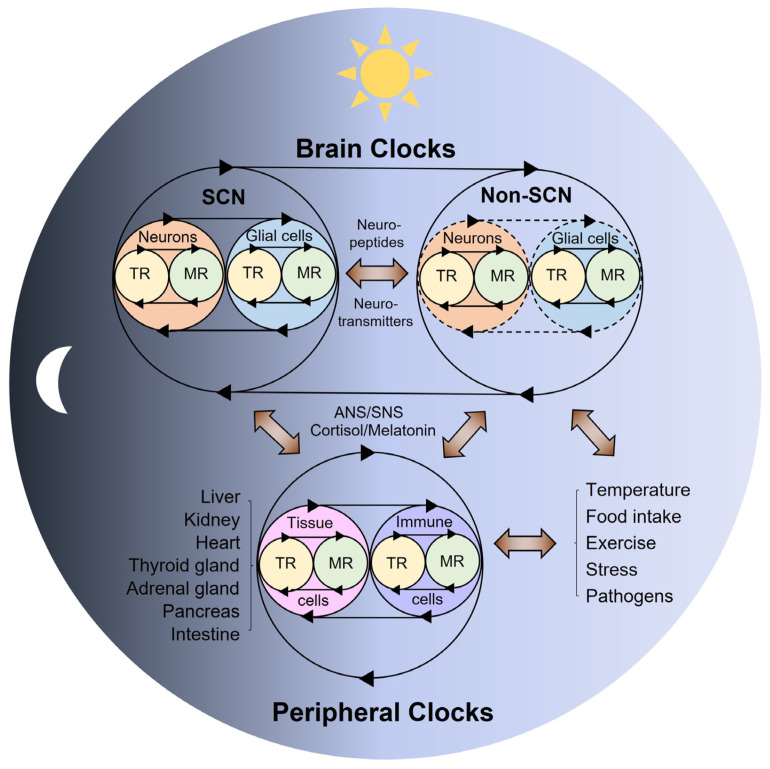
Coupled-tissue oscillators. Reciprocal crosstalk between the brain and peripheral clocks. The coupled TR and MR oscillators are thought to be commonly present across all body cells. Neurons and glial cells (e.g., astrocytes, microglia) interact to form the SCN central clock and non-SCN clocks in the brain. These autonomous brain clocks communicate with each other via neurotransmitters or neuropeptides, and with multiple peripheral tissue clocks via systemic innervations (ANS/SNS) or hormonal signals (e.g., cortisol, melatonin) in response to light–dark cycles. On the other hand, peripheral organs possess tissue autonomous clocks that can respond to non-photic physiological and environmental cues (e.g., temperature, food intake, exercise, stress) and provide feedback that influences the brain clocks via immune, metabolic, and endocrine signals. TR—transcriptional rhythms; MR—metabolic rhythms; ANS—autonomic nervous system; SNS—sympathetic nervous system.

In mammalian species, the circadian clock machinery is shared across the brain and peripheral organ systems, constituting a body-wide circadian network (Figure 2).

The intracellular oscillators, in approximately 20,000 neurons, comprise the hypothalamic suprachiasmatic nucleus (SCN), a central clock in the rodent brain. The SCN in humans has been found to contain a total number of neurons close to 100,000, though these numbers decline with age [46,47,48]. The SCN consists of different subtypes of neurons that express the neurotransmitter c-aminobutyric acid (GABA), an inhibitory neurotransmitter in the brain, alongside a range of neuropeptides such as vasoactive intestinal peptide (VIP), arginine vasopressin (AVP), and their cognate receptors (VPAC2 and AVPR1A/B) [49,50,51,52]. These GABAergic/peptidergic SCN neurons interact among themselves or with the other neurons in extra-SCN brain regions, constituting the main output pathway of the clock. Notably, a recent study has demonstrated that the VIP-VPAC2 neuropeptidergic axis plays a central role in mediating the endogenous pacemaking function of the SCN circadian circuit [53]. In line with this, the suprachiasmatic VIP neurons (SCN^VIP^) have been shown to be required for normal circadian behaviors via functional connectivity between SCN^VIP^ neurons and dorsomedial hypothalamic neurons [54]. Interestingly, recent single-cell RNA sequencing (scRNASeq) studies with mouse SCN slice revealed novel neuronal phenotypes and interaction networks involved in the central clock, including the identification of five SCN neuronal subtypes, with cluster-specific marker genes of VIP, AVP, gastrin-releasing peptide (GRP), cholecystokinin (CCK), and the cell adhesion regulator C1ql3 [55,56]. Additional scRNASeq study has also identified a subgroup of cells expressing Prokineticin2 (Prok2) and its cognate receptor (ProkR2) found to be topologically and functionally distinct pacemaking element of the central clock [57]. Thus, these studies highlight diverse cellular sub-populations within the neuropeptidergic topology of the SCN, which may differently contribute to the central pacemaker function. Notably, recent studies have shown that astrocytes harbor distinct rhythmic properties, such as anti-phasic Ca^2+^ rhythms, that direct the circadian rhythmicity of SCN neurons and behavior [58,59,60]. This work suggests the importance of bipartite intercellular communication between astrocytes and neurons in modulating SCN pacemaker functions, beyond neuronal regulation of the central clock.

Along with endogenous circadian pacemaking activity, the SCN central clock also mediates the periodic synchronization of internal body rhythms with external day and night cycles by communicating retinal light information received from the retinohypothalamic tract (RHT) to peripheral clock systems [26]. In a hierarchical organization model, the SCN master clock coordinates the circadian phases of individual subordinate clocks in other brain regions via rhythmic release of neurotransmitters and neuropeptides as well as in peripheral tissues via systemic hormonal secretion and neural innervations, thus generating rhythmic output physiology and behaviors that are in keeping with the daily changes in environment and needs [61,62,63,64]. For example, the SCN coordinates the rhythmic anti-phasic secretion of the night sleep hormone melatonin in the pineal gland and the morning stress hormone glucocorticoid in the adrenal glands via the sympathetic nervous system, ensuring daily rhythms in sleep/wake, as well as neural, metabolic, and immune functions [65,66,67]. In addition, the brain master clock controls peripheral clock functions in the heart, kidney, pancreas, lung, intestine, and thyroid glands by circadian regulation of the autonomic nervous system [68,69,70,71,72,73].

Besides SCN-driven clock entrainment, a growing number of studies have reported that non-SCN brain regions and peripheral tissues possess their own autonomous, entrainable oscillators that influence not only circadian functions in the SCN and neighboring local clocks, but also whole body rhythms via neural, hormonal, and metabolic feedback mechanisms [13,74,75,76,77,78,79,80]. The SCN receives a myriad of nonphotic input, arousal, feeding behavior, locomotor activity, immune function, blood pressure, and melatonin, which are all able to adjust and synchronize the SCN [81,82,83,84]. The SCN can receive this feedback through its large array of reciprocal neuronal connections with different hypothalamic regions, such as the arcuate nucleus (ARC), intergeniculate leaflet (IGL), nucleus tractus solitarius (NTS), dorsal raphe, and dorsomedial hypothalamus, allowing these nuclei to convey nonphotic feedback to the SCN and thus adjusting circadian rhythmicity [85,86,87,88,89].

Beyond photic entrainment, multiple physiological and environmental cues (e.g., food intake, gut microbial products, redox cofactors, metal ions, metabolic gases) control non-SCN and peripheral clock functions, which, in turn, impact the entire host clock system via neural and immune–metabolic circuits [12,22,72,90,91,92,93,94,95]. These findings suggest that systemic circadian rhythms are achieved through multi-modal regulation of tightly coupled body clocks according to daily changes that occur in the internal and external environments.

## 3. The Role of Brain Clocks in Circadian Rhythms and Disorders

The growing knowledge of cellular and tissue clock coupling mechanisms has increased our understanding of how rhythm disturbances of the SCN or locally distributed clocks within other tissues, caused by multiple physiological and environmental factors, impact circadian physiology and diseases at the whole-body level. Indeed, numerous animal studies have demonstrated pathological impacts of experimental circadian disruptions, such as surgical ablation of the SCN specific clock, whole-body knockout of core clock genes (i.e., *Bmal1*, *Per1/2*, *Cry1/2*, *Rev-erbα/β*), and experimental jet lag (chronic phase shift by altered light schedules). These studies provide ample evidence for the increased risk of sleep, mood, cardiometabolic, immune, endocrine, digestive, reproductive, premature aging, neurodegenerative, and neoplastic disorders [13,69,71,96,97,98,99,100,101,102]. Accordingly, several human health and translational studies have reported similar adverse health consequences as a result of circadian clock gene polymorphisms, sleep deprivation, night shift work, and artificial light at night (LAN) exposure [103,104,105,106].

The recent development of a tissue-specific genetic disruption tool has provided further insights into the specific roles of the SCN and its surrounding brain regions in circadian rhythm regulation. Studies of mice with genetic ablation of *Bmal1* in SCN neurons showed that peripheral clocks, as well as locomotor activity, were lost or compromised in constant darkness but remained rhythmic and synchronized to light–dark (LD) conditions [107,108]. This suggests that previously observed loss of circadian rhythms, even under LD cycles, in animals with surgical ablation of the SCN resulted from the elimination of SCN afferents [109,110,111]. In a similar study, forebrain/SCN-specific deletion of *Bmal1* in mice resulted in the immediate loss of circadian behavior as well as desynchronized and dampened circadian rhythms in peripheral tissues under conditions of constant darkness [112]. However, the loss of behavior and peripheral synchrony was rescued by LD cycles and was partially rescued by restricted feeding [112]. Moreover, targeted deletion of *Bmal1* in neurons and glia was found to exacerbate neurodegenerative pathologies and behaviors, despite the retention of intact circadian behavioral and sleep–wake rhythms under LD conditions [101]. In line with these findings, a recent study showed that the nuclear receptors Rev-erbα/β in the GABAergic (γ-aminobutyric acid-producing) in neurons of the SCN (SCN^GABA^ neurons) control the diurnal rhythm of insulin-mediated suppression of hepatic glucose production in mice, without affecting diurnal eating or locomotor behaviors during regular light–dark cycles [113]. These results suggest that the SCN central clock plays an important role in maintaining the internal synchrony of robust circadian programs in some peripheral clocks, while other peripheral clocks can be differentially modulated by light and feeding, regardless of the presence of a functional SCN central oscillator in the brain.

Beyond the SCN’s clock-modulatory functions, accumulating evidence has revealed a critical role for SCN-independent clock entrainment in circadian rhythms and disorders. Recent studies have shown that several brain regions outside the SCN (e.g., extra-SCN hypothalamic nuclei, hippocampus, lateral habenula, choroid plexus) express SCN-independent or semi-autonomous intrinsic circadian rhythms that are sensitive to fluctuations in nutrients, behavioral arousal, and hormonal signals, particularly driven by daily food intake [75,79,80]. Through ablation or genetic perturbation, these studies revealed the important roles of non-SCN clock functions in modulating downstream body activities including metabolic rate, thermogenesis, food consumption, thirst, mood, and sleep [75,79,80].

Notably, SCN-independent clocks can modulate the activity of the SCN oscillator itself. With the combined approaches of pharmacological inhibition and electrophysiological stimulation of the substantia innominata (SI) in the basal forebrain, a recent study has argued that the cholinergic basal forebrain is both a necessary and sufficient arousal input to the circadian pacemaker, mediating arousal-induced clock resetting [114]. These results may also explain why, in the absence of a functional SCN clock in the brain, behavioral rhythms, and peripheral clocks are still entrained when mice are kept under LD cycle conditions [112,115]. The SCN circuitry is thus capable of conveying rhythmicity secondarily to other oscillators. Consistent with these findings, another recent study using an in vitro tissue co-culture model revealed that the autonomous clock in the choroid plexus (CP), the brain region responsible for cerebrospinal fluid (CSF) secretion, exceeds even the SCN clock in terms of robustness, perhaps determining the circadian period of behavioral locomotor activities by speeding up the SCN clock via the circulation of CSF [75]. In a parallel study, this CP oscillator was shown to be modulated by estrogens via a hormone receptor-dependent mechanism [116]. Together, these results indicate the physiological importance of non-SCN brain oscillator functions in mediating systemic regulation and environmental entrainment of biological clock function.

## 4. The Role of Peripheral Clocks in Circadian Rhythms and Disorders

In addition to the brain network of cellular oscillators, growing evidence suggests that self-driving clocks in peripheral tissues are critical for the systemic homeostasis of circadian physiology. An approach that has been applied extensively in this context is tissue-specific disruption of a core clock component, for instance, the *bmal1* locus, paired with assessment of physiological and endocrinological functions of that tissue. Table 1 lists some examples of studies in which this approach has yielded novel insights into the function of peripheral clocks in physiology and endocrinology.

For example, in a prevailing model, the robust daily rhythm of glucocorticoid (GC; corticosterone (CS) in rodents and cortisol in primates) has generally been attributed to SCN-mediated modulation of the hypothalamic–pituitary–adrenal (HPA) stress response axis and sympathetic innervation of the GC secretory adrenal gland for the light entrainment of other peripheral clock systems [65,66,129]. On the other hand, Kim and colleagues have shown that the adrenal gland has its own autonomous clock that functions independently of the SCN, and that adrenal-specific disruption of Bmal1 dampens the circadian CS rhythm, impairing behavioral rhythmicity and gene expression cycling in several peripheral organs [76,118]. These findings correspond with another study showing that the adrenal-intrinsic oscillator controls a gating mechanism involved in circadian sensitivity to adrenocorticotropin (ACTH) produced by the pituitary gland [119,130].

In another peripheral clock study, liver-specific *Bmal1* deletion was shown to increase glucose clearance and hypoglycemia restricted to the fasting phase as a result of arrhythmic gene expression of glucose-related genes [120]. The systemic impact of the liver clock was further demonstrated in additional studies showing that dual *Rev-erbα/β* knockout specifically in the liver leads to increased circulating glucose and triglyceride levels, a reduction in free fatty acids (FFAs), as well as altered transcriptional, metabolic, and behavioral rhythms, compared with controls [131,132,133]. Notably, a more recent study revealed that circadian oscillations persist in the livers of mice devoid of an SCN or oscillators in cells other than hepatocytes, corroborating the independent nature of the liver clock [78].

Similar to liver cells, adipocytes harbor their own circadian clock. Disruption of this clock via adipocyte-specific deletion of *Bmal1* results in obesity in mice, with a shift in the diurnal rhythm of food intake and energy expenditure, suggesting a central role for adipose tissue as an integrator of organismal energy balance [117]. In support of this idea, a recent study using an adiponectin (ADIPOQ)-deficient mouse model showed that this adipokine, which is produced primarily in adipose tissue to modulate glucose levels as well as fatty acid breakdown, regulates diurnal feeding rhythms through clocks in energy regulatory centers (e.g., arcuate nucleus (ARC)) of the mediobasal hypothalamus (MBH) [134]. The physiological implications of these mechanistic studies in animals are further underscored by previous human studies showing that in obese patients, ADIPOQ blood levels decline and the diurnal rhythms of ADIPOQ release are dampened [135,136]. On the other hand, another endocrine study showed that ablation of the pancreatic β cell-autonomous clock in adult mice caused severe glucose intolerance along with altered insulin secretion [121]. Interestingly, the O’Neill group recently provided evidence of the role of insulin as a systemic timing cue to entrain circadian rhythms throughout the body by selective induction of the period clock proteins, with further demonstration of the circadian disruption recapitulated by mistimed insulin in cell and animal models [137].

Importantly, growing evidence suggests an independent physiological role for the skeletal muscle clock. Recently, Ehlen et al. reported that muscle-specific Bmal1 disruption and restoration in *Bmal1*-KO mice led to sleep disturbances and recovery, respectively; however, brain-specific restoration of BMAL1 expression did not rescue BMAL1-dependent sleep phenotypes, indicating that the skeletal muscle clock is both necessary and sufficient for normal sleep regulation [122]. In line with these findings, a subsequent study indicated that muscle-specific loss of BMAL1 is associated with metabolic inefficiency, impaired muscle triglyceride biosynthesis, and the accumulation of bioactive lipids and amino acids [123]. More recently, skeletal muscle was also shown to exhibit diurnal variations in muscle growth and metabolism, independent of locomotor activity [138]. Thus, it can be speculated that rhythmic metabolic signals from the skeletal muscle clock impact systemic energy homeostasis, thus regulating brain activity and sleep function.

Notably, emerging transcriptomic and metabolomic studies have reported that lung adenocarcinoma as well as pulmonary inflammation (e.g., asthma) can reprogram the circadian homeostasis of local and systemic physiology, leading to metabolic and sleep disturbances [139,140]. In line with the idea that local organ disease impacts circadian rhythms, a recent study using a chronic kidney disease (CKD) mouse model reported that kidney-specific clock disruption by CKD leads to unstable circadian behavioral rhythms, which may explain the sleep disturbances that are known to be associated with kidney failure [77]. It is assumed that the rhythmic readjustment of fluid homeostasis by the kidney affects the SCN clock, thereby altering circadian rhythms at the behavioral level [77]. Overall, the current evidence suggests that brain clocks as well as multiple regulatory feedback networks in the periphery contribute to circadian physiology and pathophysiology (Figure 2).

## 5. The Role of Feeding in Circadian Rhythms and Disorders

Besides the systemic impact of local clock dysfunction, growing evidence has emphasized the chrono-physiological importance of non-photic regulation of the body clock network. Several early studies showed that timed food administration affects peripheral clocks without altering the SCN [141,142]. However, recent comprehensive metabolomics studies demonstrated that nutritional challenges, such as a high-fat diet, metabolically reprogram the circadian rhythmicity of distinct brain areas, including the SCN, and peripheral clocks, thus altering the system-wide synchrony of the internal body clocks [38,143,144]. In addition to an unbalanced diet, short-term feeding at the wrong time is reported to be sufficient to desynchronize peripheral clocks and induce obesity along with hyperphagia, physical inactivity, and metabolic disorders in mice [145]. These observations from animal models are resonant with several human studies, reporting that food intake during the circadian evening and/or night is associated with increased body fat and obesity and reduced effectiveness of weight loss therapy in both adults and children [146,147,148,149]. Besides the metabolic impact, mistimed feeding was also shown to result in dramatic deficits in hippocampus-dependent learning and memory by reducing cyclic AMP response element binding protein (CREB)-mediated long-term potentiation in the hippocampus [150]. Moreover, a recent population-based case–control study reported that mistimed eating patterns increase the risk of breast and prostate cancer [151].

Notably, an increasing number of studies has revealed that the intestinal microbiota in both mice and humans exhibit diurnal oscillations in composition and activity, and that these rhythms are impaired by the ablation of host molecular clock components or the induction of jet lag, leading to gut dysbiosis and altered feeding rhythms [37,152,153,154]. The potential systemic impact of a dysregulated microbiome was further shown in a subsequent study indicating that the diurnal rhythmicity of the microbiota drives the global programming of host circadian transcriptional, epigenetic, and metabolite oscillations [90]. Interestingly, Chambon and colleagues from the analysis of large intestine showed that depletion of microbiota profoundly disrupts circadian clock in intestinal epithelial cells (IECs), leading to altering corticosteroids levels and consequent metabolic disorders [155]. This suggests a reciprocal influence between the gut epithelium of the host and the microbiota for each other’s physiology that contributes to additional system-wide cyclic homeostasis. In line with these studies, Kuang et al. recently reported that cyclically induced expression of histone deacetylase 3 (HDAC3) in gut epithelial cells mediates the diurnal programming of the host’s metabolism by the intestinal microbiota, the lack of which leads to diet-induced obesity [156]. Given the systemic modulation of both brain and peripheral clock functions by gut microbiome metabolites [157], it is plausible that the above pathological impact of mistimed feeding is associated, in part, with dysregulated gut-microbiome products. Together, these findings underscore the physiological and pathological significance of peripheral entrainment of host circadian systems in response to multiple internal and external cues.

## 6. The Role of Metabolic Cues in Circadian Rhythms and Disorders

Emerging studies suggest that multiple metabolic or nutritional components, such as redox cofactors, gases, and ions, are not only circadian-regulated components, but can also act as timing cues to regulate circadian physiology via reciprocal feedback mechanisms (Figure 1). Descriptions of some of these factors and their roles in circadian rhythms and disorders are detailed below.

### 6.1. NAD(P)/NAD(P)H

Nicotinamide adenine dinucleotide (NAD) (including NAD+ and NADH) and nicotinamide adenine dinucleotide phosphate (NADP) (including NADP+ and NADPH), via their electron transfer functions, are fundamental mediators of various biological processes, including energy metabolism, mitochondrial functions, anti-oxidation/generation of oxidative stress, gene expression, immunological functions, and aging [158]. Several lines of evidence suggest that NAD(P)/NAD(P)H are circadian-controlled or are circadian controlling factors. Previous studies have shown that intracellular NAD+ levels cycle with a 24 h rhythm driven by CLOCK:BMAL1-dependent circadian expression of nicotinamide phosphoribosyltransferase (NAMPT), a rate-limiting enzyme in the NAD+ salvage pathway [159,160]. In turn, the NAD(P)/NAD(P)H ratio impacts circadian rhythms at a molecular level both directly and indirectly. In an early in vitro study, Rutter et al. showed that the reduced forms of the redox cofactors NADH and NADPH strongly enhance DNA binding of CLOCK/Neuronal PAS Domain Protein 2 (NPAS2):BMAL1 heterodimers, whereas the oxidized forms of the cofactors (NAD+ and NADP+) inhibit binding [161]. In later studies, increasing NADH levels by inhibiting NAMPT was found to promote oscillation of the clock gene *Per2* by releasing CLOCK:BMAL1 from suppression by SIRT1, an NAD+-dependent protein deacetylase that was previously shown to counteract CLOCK acetylation and suppress CLOCK-BMAL1-mediated transactivation [160,162]. On the other hand, a recent study showed that NADPH depletion by pharmacological inhibition of the pentose phosphate pathway (PPP), an NADPH-generating pathway that is tightly connected to glycolysis, leads to the activation of CLOCK/BMAL1 by the histone acetyltransferase p300 [94]. These data suggest that the NAD(P)/NAD(P)H redox ratio can regulate CLOCK:BMAL1-dependent circadian function by affecting dimerization or epigenetic cofactor recruitment. Notably, a more recent study showed that NAD+ activation of SIRT1 rejuvenates circadian rhythms in old mice through inhibition of the clock repressor PER2 by protein deacetylation, which controls its phosphorylation-mediated nuclear entry and stability [163]. This finding is reminiscent of a previous result showing that SIRT1 binds to CLOCK-BMAL1 in a circadian manner and promotes the deacetylation and degradation of PER2 [164]. Given the emerging role of NAD+/SIRT1 pathways in fasting diet and anti-aging pathways [165], these results provide important mechanistic insights into the close relationship between circadian rhythms and aging-related pathogenic processes.

### 6.2. Heme

A growing body of evidence indicates that circadian rhythms can also be regulated by additional redox-active compounds including heme, its breakdown product CO gas, as well as NO gas. Heme is an iron-containing porphyrin that has several functions, from oxygen transport to electron transfer and catalysis [166]. In early studies, heme was shown to be rhythmically controlled by cyclic synthesis and degradation, mediated by the circadian-regulated enzymes aminolevulinic acid synthase (ALAS1) and heme oxygenase (HO), respectively [167]. It has also been described to be a ligand of REV-ERBα that represses its transcriptional activity by recruiting co-repressors, thereby coordinating circadian and metabolic pathways [168,169]. Heme has also been shown to bind to CLOCK or NPAS2 in vitro, mediating the reduced DNA binding of CLOCK/NPAS2:BMAL1, induced by carbon-monoxide (CO) [170,171]. Furthermore, a structural and functional study revealed that heme directly binds to CLOCK protein and disrupts the binding of this circadian activator to its E-box DNA target [172]. These findings nicely correspond with a previous functional study showing that heme dose-dependently and reversibly dampens luminescence rhythms in SCN explants from mice expressing an E-box-containing *Per2* promoter-driven luciferase (PER2::LUC) reporter [173].

### 6.3. Carbon Monoxide (CO)

Carbon monoxide (CO) and nitric oxide (NO; see below) are gasotransmitters, endogenously generated, membrane-permeable gasses that impact on cellular signaling pathways. In addition to the role of heme in molecular clock function, Klemz et al. recently reported that CO, a natural by-product gas of heme breakdown, suppresses circadian transcription by attenuating CLOCK-BMAL1 binding to target promoters [174]. Conversely, suppression of CO production was shown to lead to a global upregulation of circadian gene expression and dysregulated glucose metabolism in mouse hepatocytes [174]. Further analysis showed that pharmacological inhibition or genetic depletion of CO-producing heme oxygenases abrogated normal daily cycles in mammalian cells and in *Drosophila* (fruit flies), suggesting the requirement for daily cycles of CO to maintain normal circadian rhythms as well as circadian metabolic outputs [174]. The crucial role of CO in circadian rhythms was further confirmed by a later study showing that selective removal of endogenous CO by administration of hemoCD1, a highly selective CO scavenger, considerably disrupts CLOCK/NPAS2:BMAL1-dependent rhythmic expression of clock genes in mice [175]. Notably, against the common notion of CO as a metabolic waste product or toxic poison, this circadian gasotransmitter is becoming an emerging therapeutic target for neuroprotection as well as for treating inflammation, cancer, and sickle cell disease [176,177]. In line with this notion, recent studies have shown that CO exerts a protective effect on neuronal or ischemic injuries by preserving or enhancing circadian rhythms [178,179].

### 6.4. Nitric Oxide (NO)

As a gasotransmitter synthesized by three enzymes (endothelial, inducible, and neuronal nitric oxide synthases) with varying patterns of tissue-specific expression, NO plays an important role as a protective mediator in the cardiovascular and neurovascular systems [180]. Accumulating evidence suggests that NO is a rhythmically controlled gas molecule that can influence circadian rhythms and diseases. In an early study, circadian variation of eNOS activity and cytosolic protein content was reported in several regions of the rodent brain, such as the cerebellum, brainstem, hypothalamus, and hippocampus, that reach acrophase in the active phase [181]. A later study using in vivo microdialysis showed that the extracellular concentration of nitrite (NO_2_^−^), an oxidized product of NO, exhibited persistent circadian rhythms in the dorsal rat SCN under constant darkness as well as during the LD cycle [182]. This suggests that the endogenous rhythm of NO is regulated independently of photic inputs into the SCN. Indeed, in other studies, the endogenous rhythms of eNOS activity in brain, kidney, testis, and lungs, as well as plasma NO levels, displayed 24 h rhythms under either LD or continuously lit conditions [183,184]. Furthermore, the rhythms of eNOS activity and expression were shown in the hippocampus of the pigeon and in isolated chick choroid, suggesting the evolutionary conservation of NO as a circadian-regulated signaling molecule [185].

The functional relevance of NO synthesized by endothelial nitric oxide synthase (eNOS) in circadian rhythmicity was shown in a recent study reporting that a time-of-day variation in vascular smooth muscle contractility occurs in phase with the daily fluctuation of *eNOS* mRNA levels [186]. Further evidence has also suggested a circadian mechanism underlying the NO cycle. Interestingly, leptin, a hormone that mainly acts to regulate appetite and fat storage, was found to cycle in phase with the NO cycle in adipocytes and also stimulated NO release from the hypothalamus and anterior pituitary gland in rats [187]. This data suggests that the circadian release of leptin contributes to the systemic NO cycle. In a later study, it was shown that mutation of the *Per2* gene in mice led to aortic endothelial dysfunction involving mechanisms that included the decreased production of NO [188]. In addition, *Bmal1* KO resulted in uncoupling of eNOS from its cofactor tetrahydrobiopterin (BH4) and increased superoxide at the expense of NO, contributing to endothelial dysfunction [189], indicating the physiological importance of clock genes in the regulation of NO. In line with the animal studies, a study in humans showed day–night variations in blood levels of NO in both healthy adults and subjects with type II diabetes; however, higher concentrations of NO were observed in the diabetic subjects compared with the healthy ones [190]. Thus, it was reasoned that higher concentrations of NO may result in increased vascular wall uptake of lipoproteins in diabetic subjects, who are at greater risk than healthy subjects for developing diffuse atherosclerosis [190].

Despite the conserved NO rhythmicity, it is controversial whether NO can regulate circadian rhythms. Genetic knockout model studies showed no significant changes in phase-shift locomotor activity and circadian blood pressure in *eNOS^−/−^* mice compared with wild-type mice under LD or constantly dark conditions [191,192], suggesting that eNOS may not be critical for photic entrainment in mice. Upregulation of another of the three NOS isoforms may have compensated for eNOS deficiency in this model. However, a later study showed that NO upregulated the circadian expression of *Per* genes via cAMP response element- dependent and E-box enhancer element-dependent pathways [193]. This is reminiscent of previous studies showing that exogenous NO potentiated photic phase shifts of circadian rhythms, while the infusion of a NOS inhibitor disrupted the rhythms of water intake [194]. Notably, along with impaired clock gene expression and circadian variation in blood pressure, eNOS activity was shown to be markedly decreased during the daytime in aged animals; however, treatment with an NO donor significantly improved these impairments [193]. Considering the emerging role of NO as an antioxidant and anti-aging molecule, this data suggests that NO can prevent or mitigate pathological disruption of circadian rhythms. Related to this, a recent *Drosophila* model study showed that fly mutants lacking NOS had behavioral arrhythmia in constant darkness, although molecular clocks in the main pacemaker neurons were unaffected [195]. The rhythm disruptive phenotypes of these mutants were found to be due, in part, to malformation of the neurites of small ventral lateral clock neurons (s-LNvs), the main circadian pacemaking cells in the fly brain [195]. This study also identified perineurial glia, one of the two glial subtypes that form the blood–brain barrier, as the major source of NO regulating circadian locomotor output [195]. Taken together, these findings suggest an important role for NO as a systemic signaling molecule in the normal maintenance of circadian physiology and behavior.

### 6.5. Oxygen (O_2_)

Similar to CO, low oxygen (hypoxia) has been suggested as a time cue for entraining molecular clocks. Hypoxia-inducible transcription factors (HIFs), which respond to low oxygen levels and induce its re-supply, interact with BMAL1 via their shared dimerization domains and DNA-binding motifs to mediate the cross-talk between the circadian and hypoxia signaling pathways [196]. In mouse brain, it was shown that hypoxia induces the expression of the circadian genes *Per1* and *Clock* [197]. In subsequent human studies, hypoxia was shown to alter the circadian oscillation of multiple physiological processes, such as body temperature, metabolic rate, cortisol, and melatonin release, leading to sleep disturbances [198,199,200]. Recently, Adamovich and colleagues documented daily rhythms of tissue oxygenation and oxygen consumption and showed that oxygen cycles, as well as hypoxic conditions, reset cellular and behavioral rhythms in an HIF1α-dependent manner [201,202]. Thus, the authors suggested that oxygen, via HIF1α activation, is a resetting cue for circadian clocks and proposed oxygen modulation as a therapy for jet lag. In this regard, since oxygen delivery has been implemented as an effective therapy for patients with COVID-19 pneumonia [203], it would be interesting to take into account the potential effects of oxygen treatment on circadian physiology and disease outcomes [204].

### 6.6. Carbon Dioxide (CO_2_)

Notably, Adamovich and colleagues recently showed that the daily rhythms of carbon dioxide (CO_2_) release and oxygen consumption are tightly coupled and circadian clock-controlled, and that time-restricted feeding restores their altered rhythmicity in the clock-deficient mice [201]. Interestingly, further results showed that oxygen rhythms were dominated by activity, while CO_2_ oscillations were shifted and aligned with food intake [201]. Moreover, changes in CO_2_ levels altered clock gene expression and phase-shifted the circadian rhythms [201]. Collectively, these results suggest that CO_2_ rhythms is not only feeding-regulated but also mediates phase resetting of peripheral clocks upon feeding.

### 6.7. Hydrogen Peroxide (H_2_O_2_)

Emerging studies have suggested that hydrogen peroxide (H_2_O_2_) is also a circadian time cue. H_2_O_2_ is an oxidizing agent through its easy release of a single oxygen atom, which is very reactive and toxic to most living organisms, that occurs as a consequence of the oxidation of proteins, membrane lipids, and DNA. In eukaryotic species, H_2_O_2_ is produced as a by-product of oxygen combustion in mitochondria and is the most stable and diffusible of the reactive oxygen species (ROS) [205]. In the cyanobacterium *Microcystis aeruginosa*, H_2_O_2_ was found to alter the expression rhythms of circadian clock and metabolic genes during the LD cycle [206]. Another study in *Drosophila* showed that H_2_O_2_ stimulates activity and alters daily locomotor rhythms, suggesting its role as a systemic circadian cue [207]. In line with these studies, a recent study by Fei Pei and colleagues showed that H_2_O_2_ exhibits cell-autonomous rhythms that induce periodic oxidation of the CLOCK protein as well as heterodimerization of CLOCK with BMAL1 followed by DNA binding, thus enhancing the amplitude of cyclic gene expression that affects the daily rhythms of behavior in mice [208]. This evidence suggests that H_2_O_2_ acts as a potent timing signal by directly interacting with the core clock machinery, thus mediating the close connection of daily redox and circadian cycles. Notably, multiple studies have shown that ROS levels are elevated and increase the severity of several diseases with genetic (e.g., *Bmal1* KO), environmental (e.g., sleep deprivation, forced chronic jet lag, mistimed feeding, alcohol intake, psycho-social stress, etc.), or pathological (e.g., aging, cancer) disruption of the circadian clock [98,209,210,211]. Beyond the roles of ROS as prime modulators of cellular, metabolic, and immune dysfunctions, these findings provide important mechanistic insights into how ROS act as direct core clock-controlling molecules, mediating the vicious cycle between circadian disruption and disease processes.

### 6.8. Hydrogen Sulfide (H_2_S)

A growing body of evidence suggests that another gasotransmitter, hydrogen sulfide (H_2_S), is a potential circadian timing modulator. H_2_S is a gas that is generated endogenously by cystathionine β-synthase (CBS), cystathionine γ-lyase (CSE), and 3-mercaptopyruvate sulfurtransferase (MPST) [212], and serves as a stimulator of electron transport in mammalian mitochondria by acting as an electron donor. Though previously known only as a toxic gas and environmental hazard, it has been demonstrated that H_2_S mediates a wide range of physiological processes, including blood vessel relaxation, cardioprotection, neurotransmission, neuroprotection, and insulin secretion [213,214]. Interestingly, it has been reported that exogenous H_2_S has a protective effect on maintaining the circadian rhythm of clock genes in isolated hepatocytes by changing the NAD+/NADH ratio and enhancing the activity of SIRT1 [215]. However, a recent study showed that elevating H_2_S levels using GYY4137, an exogenous H_2_S donor, decreased *Per2* expression, whereas depleting H_2_S by pharmacological inhibition of CBS increased the general expression and dynamics of several clock genes in serum-shocked NIH3T3 cells, suggesting that the CBS/H_2_S pathway may participate in circadian clock regulation. Related to this, it was recently reported that CBS physically interacts with CRY1, augmenting CRY1-mediated repression of the CLOCK:BMAL1 complex and shortening the circadian period in cells, while CRY1 stabilizes CBS enzymatic function. Thus, multiple mechanisms contribute to the bidirectional regulation of the CBS/H_2_S pathway and the circadian clock. On the other hand, another study showed that plasma H_2_S concentration exhibits diurnal fluctuations, along with time of day MPST activity and H_2_O_2_ concentration, in mice [216]. The diurnal H_2_S fluctuation appeared to be H_2_O_2_-dependent because it disappeared when the mice were treated with dithiothreitol (DTT), a reductant used to clean ROS [216]. Thus, it was postulated that the rhythmic changes in redox-sensitive MPST activity modulated by plasma H_2_O_2_ oscillation led to the diurnal fluctuations of plasma H_2_S [216] However, a recent study showed that H_2_S increases the production of NADPH oxidase-dependent H_2_O_2,_ suggesting a dynamic regulatory relationship between H_2_S and H_2_O_2_ synthesis/activity cycles [217]. Notably, increasing evidence has shown that treatment with exogenous H_2_S prevents or reverses aging and age-related pathologies [165,218]. In line with this data, it has been reported that H_2_S restores the diurnal variation in cardiac function that is disrupted in aging mice by reducing oxidative stress [219]. Taken together, these results suggest a potential clock-modulatory role of H_2_S that can be leveraged to improve both circadian rhythms and disorders.

### 6.9. Minerals and Metal Ions

Minerals and metal ions are fundamental elements that can mediate multiple physiological processes including neural, metabolic, and immune functions. Dysregulated production of or environmental exposure to certain mineral or metal ion species can cause sleep and psychiatric disturbances, metabolic and endocrine disorders, or neurological degeneration [93]. It has been well-documented that several ions display evolutionarily conserved rhythms in cellular concentration and transport across several species [220]. For example, calcium (Ca^2+^) has robust circadian and diurnal rhythms in plants, flies, and mice to modulate temporal physiology and behaviors [221,222,223,224]. Notably, an increasing number of studies in the plant *Arabidopsis thaliana* report that intracellular transport of copper (Cu^2+^) ion is clock-controlled, and, in turn, endogenous Cu^2+^ cycles as well as exogenous Cu^2+^ influence the circadian clock by regulating the expression of core clock components and clock output genes [220,225,226,227]. These results suggest the ubiquitous nature of reciprocal feedback regulation between the circadian clock and rhythmic ion cycles. Likewise, in early human studies, several metal ions, including Cu^2+^, zinc, lead, and mercury, were observed to exhibit circadian variation in plasma and urine [228,229,230]. These rhythms were, in part, attributed to circadian renal functions such as daily rhythms in glomerular filtration and reabsorption by the distal tubule and collecting duct during the night and morning hours [230]. In line with this, circadian profiles of calcium (Ca), magnesium (Mg), iron (Fe), Cu^2+^, zinc (Zn), lead (Pb), cadmium (Cd), cobalt (Co), chromium (Cr), and nickel (Ni) were observed in the urine of healthy, middle-aged men [231].

The functional relevance of ion cycles in circadian physiology, particularly in the brain, has been further suggested by recent animal studies. Using fly and mouse models, Flourakis et al. reported conserved ion cycles for circadian clock control of membrane excitability [232]. Their results showed that during the morning/day, sodium influx mediated by the sodium leak channel (NALCN) was elevated while resting potassium (K^+^) currents were reduced, depolarizing neurons to promote elevated firing rates. Conversely, sodium leak was low and resting K^+^ ion currents were elevated during the evening/night, hyperpolarizing cells, and suppressing firing rates [232]. Moreover, the rhythmic NA/NALCN current in fly and mouse clock neurons was shown to be driven by clock-controlled expression of NCA localization factor 1 (*Nlf-1*), a gene previously known to regulate neuronal excitability and rhythmic behaviors in *C. elegans* and mice [232,233]. A similar, subsequent study showed that anti-phasic changes in the composition of brain interstitial ions (K^+^ versus Ca^2+^, Mg^2+^) control the sleep–wake cycle [234]. Indeed, in vivo recording in mouse brain showed that arousal was linked to the elevation of interstitial K^+^ levels concomitant with decreases in interstitial Ca^2+^ and Mg^2+^ levels as well as extracellular volume. Conversely, natural sleep and anesthesia reduced K^+^ levels while increasing Ca^2+^ and Mg^2+^ levels along with extracellular volume [234].

On the other hand, accumulating evidence suggests that rhythmic changes in ion levels can directly influence circadian physiology and behavior. For example, Kim and colleagues showed, using in vitro cellular models, that Ca^2+^-dependent signaling pathways mediate the resetting of the circadian clock by immediate early induction of *Per* genes via rapid CLOCK/BMAL1 heterodimerization and nuclear localization [28,235]. This partly explains how intracellular Ca^2+^ generates and maintains circadian rhythms in the mouse SCN and behavioral rhythms in flies [224,236,237,238]. As with Ca^2+^, Cu^2+^ was also found to induce nighttime phase shifts of the SCN clock in a mitogen-activated protein kinase (MAPK)-dependent manner [239]. In line with this, Feeney et al. showed that intracellular Mg^2+^ exhibits robust cell-intrinsic circadian rhythms and acts as a cell-autonomous timekeeping factor to determine circadian rhythms of gene expression, ATP-dependent energy expenditure, and mTOR-mediated translation in both a human cell line and a unicellular alga [240]. Conserved circadian function of Mg^2+^ was further suggested in recent studies by the Sehgal group that reported that the magnesium cycle can mediate circadian rhythms in the permeability of the blood–brain barrier (BBB) and xenobiotic efflux in flies, mice, and humans [241,242,243].

In addition to their rhythmic neuro-modulatory functions, ionic species have been shown to regulate processes in a variety of other body systems. A recent study using electrophysiological and pharmacological approaches revealed that rhythmic K^+^ transport regulates the circadian clock in human red blood cells in the absence of conventional transcription cycles [244]. Interestingly, it has also been suggested that some dietary ions can reset circadian rhythms, influencing metabolic health and disease treatment. For example, selenium (Se), specifically known for its anti-oxidant, anti-inflammatory, and anti-viral activities, was shown to upregulate the expression of *Bmal1* in mice, leading to a significant increase in the resistance to toxicity induced by chemotherapeutic agents [245]. Moreover, another study showed that dietary iron controls circadian glucose metabolism by regulating heme-mediated interaction of REV-ERBα with its corepressor nuclear receptor corepressor 1 (NCOR) [246]. In more recent work, Gizowski et al. reported that systemic injection of salt (NaCl) solution can reset the SCN clock via salt-sensitive neurons in sodium-sensing organum vasculosum lamina terminalis (OVLT) in the brain, inducing phase-advance in circadian locomotor rhythms in mice [247]. Overall, these findings underscore the broad molecular and systemic impacts of nutrient ions on circadian rhythms and diseases.

## 7. Conclusions

In recent decades, extensive chronobiological research has expanded our understanding of the functional roles and mechanisms of the circadian clockwork in human health and diseases. Thus, research trends in chronobiology have undergone a paradigm shift in many ways, particularly changing from hierarchical models to more integrated ones for understanding the circadian clock mechanism. In this regard, the overall evidence points to bidirectional crosstalk between transcriptional and metabolic rhythms, neuronal and glial clocks, SCN and non-SCN clocks, as well as brain and peripheral clocks (Figure 1, Figure 2). Our growing knowledge of the interactive nature of clock regulatory systems is expected to not only diversify our understanding of circadian physiology and pathophysiology but also increase our capacity to harness chronobiological knowledge to improve the prevention and treatment of multiple circadian-related disorders.

## Figures and Tables

**Table 1:** A sampling of studies on the effects of tissue-specific Bmal1 ablation.

Cell type of Selective Bmal1 Ablation	Promoter Controlling Cell-Specific Manipulation	Rhythm(s) Eliminated	Effect on Circadian Behavior	Clinical Manifestation	Citation Number
Adipocyte	Adipocyte protein 2 (aP2) gene promoter	Adiponectin	Shift in the diurnal rhythm of food intake and energy expenditure	Obesity	[117]
Adrenal	Melanocortin 2 Receptor (MC2R) gene promoter, Aldosterone synthase (AS) gene promoter data	Circulating corticosterone, ACTH sensitivity	Attenuated behavioral rhythmicity	Hyperadrenocorticism	[76,118,119]
Hepatocyte	Abumin (ABL) gene promoter	Glucoregulatory genes	None	Increase glucose clearance and hypoglycemia restricted to the fasting phase	[120]
Pancreatic β cell	Pancreatic Additionally, Duodenal Homeobox 1 (PDX1) gene promoter	Insulin secretion	None	Insulin resistance	[121]
Skeletal muscle	Human α-skeletal actin (HSA) gene promoter	Muscle growth and metabolism	Sleep disturbance	Metabolic inefficiency and impaired muscle triglyceride biosynthesis	[122,123]
Renal	Kidney-specific cadherin (KSP-Cad) gene promoter	None	None	Altered the plasma metabolome, lowered blood pressure in male mice	[124,125]
Intestine	Villin (VIL1) gene promoter	None	None	Prevents obesity induced by high-fat feeding	[126]
Cardiomyocyte	Myosin heavy chain α (MHCα) gene promoter	Circadian gene expression in heart	None	Diastolic dysfunction, Impaired resolution of inflammation, Reduced life span	[127,128]

## Data Availability

No new data were created or analyzed in this study.
